# Exploring gut microbiota’s role in rheumatic valve disease: insights from a Mendelian randomization study and mediation analysis

**DOI:** 10.3389/fimmu.2024.1362753

**Published:** 2024-06-04

**Authors:** Xiwei Chen, Guangwen Hu, Dong Ning, Daxin Wang

**Affiliations:** ^1^ The Hospital Affiliated to Medical School of Yangzhou University (Taizhou People's Hospital), Taizhou, Jiangsu, China; ^2^ Department of Physiology, Human Biology Building, School of Medicine, National University of Ireland (NUI), Galway, Ireland

**Keywords:** gut microbiota, mediation analysis, Mendelian randomization, rheumatic valve disease, immune cell, estradiol

## Abstract

**Background:**

Investigating the relationship between gut microbiota and Rheumatic Valve Disease (RVD) is crucial for understanding the disease’s etiology and developing effective interventions. Our study adopts a novel approach to examine the potential causal connections between these factors.

**Methods:**

Utilizing a two-sample Mendelian Randomization (MR) framework, we incorporated a multi-variable MR (MVMR) strategy to assess the mediatory mechanisms involved. This approach involved analyzing data from the MiBioGen consortium for gut microbiota and the FinnGen for RVD, among other sources. Instrumental variables (IVs) were carefully selected based on rigorous MR principles, and statistical analysis was conducted using bidirectional two-sample MR, such as inverse variance-weighted (IVW), weighted median, MR-Egger regression and MR Steiger Test methods. The MR-PRESSO strategy was employed for outlier detection, and MVMR was used to untangle the complex relationships between multiple microbiota and RVD.

**Results:**

Our analysis highlighted several gut microbiota classes and families with potential protective effects against RVD, including *Lentisphaerae, Alphaproteobacteria*, and *Streptococcaceae*. In contrast, certain genera, such as *Eubacterium eligens* and *Odoribacter*, were identified as potential risk factors. The MVMR analysis revealed significant mediation effects of various immune cell traits and biomarkers, such as CD4^-^CD8^-^ T cells, CD3 on Terminally Differentiated CD8+ T cell and Pentraxin-related protein PTX, elucidating the complex pathways linking gut microbiota to RVD.

**Conclusion:**

This study underscores the intricate and potentially causal relationship between gut microbiota and RVD, mediated through a range of immune and hormonal factors. The use of MVMR in our methodological approach provides a more comprehensive understanding of these interactions, highlighting the gut microbiota’s potential as therapeutic targets in RVD management. Our findings pave the way for further research to explore these complex relationships and develop targeted interventions for RVD.

## Introduction

Rheumatic Valve Disease (RVD), a chronic heart condition often originating from rheumatic fever, is a significant global health challenge, particularly in regions with limited healthcare resources (1). RVD is primarily believed to result from an autoimmune response triggered by antigenic mimicry. This mimicry occurs between certain surface proteins of group A streptococci and human cell surface antigens, particularly in genetically predisposed individuals (2). The bacterial proteins resemble elements of human cardiac myosin, including the N-acetyl glucosamine carbohydrate epitope and spiral M protein (3). This resemblance activates CD4^+^ T cells, B cells, and macrophages, which mistakenly target the body’s own cells (4). Acute rheumatic fever, affecting approximately 0.3–3% of individuals infected with group A streptococci, is characterized by transient inflammatory tissue damage, usually resolving within weeks to months. Initial valvular involvement is often minimal to moderate. However, permanent valvular damage and chronic rheumatic disease develop in about 30–45% of these patients. Chronic rheumatic disease is marked by continuous heart tissue inflammation even in the absence of bacterial presence (5).

The risk of developing acute rheumatic fever or long-term Rheumatic Heart Disease (RHD) hinges on three key factors: the specific bacterial strain, the genetic predisposition of the host, and abnormal immune responses of the host (6, 7). In this context, the role of the gut microbiota, the diverse community of microorganisms residing in the human gastrointestinal tract, has emerged as a subject of interest. Recent studies have begun to shed light on the potential influence of gut microbiota on cardiovascular health. For instance, research has indicated that specific microbial compositions can contribute to systemic inflammation, a known risk factor for various cardiovascular diseases (8). Shi and colleagues’ study on RHD patients revealed altered gut and oral microbiota, potentially influencing RHD pathogenesis (9). The study found increased levels of *Bifidobacterium* and Eubacterium, and decreased levels of *Faecalibacterum* and *Bacteroides* in RHD patients compared to controls, suggesting these changes might worsen the disease by affecting the mitral valves.

This study employs Mendelian randomization (MR) to explore the causal relationship between gut microbiota composition and the risk of RVD. MR analysis uses genetic variants from Genome Wide Association Study (GWAS) as instrumental variables to assess causality, thus reducing the biases inherent in traditional observational studies (10).

It relies on the principle that these genetic variants are randomly inherited, thus acting like a natural randomized controlled trial (11). This randomness helps to avoid confounding factors that usually affect observational studies. If a genetic variant linked to a risk factor is also associated with a health outcome, it suggests a causal relationship between the risk factor and the outcome. MR provides a more robust approach to infer causality compared to traditional epidemiological methods. By leveraging genetic markers associated with microbiota profiles, this approach aims to determine whether alterations in gut microbiota are a contributing factor in RVD pathogenesis or simply an association.

Through this Mendelian randomization analysis, our objective is to provide robust, causal evidence of the role played by gut microbiota in the development of RVD. The study extends beyond analyzing microbiota composition by also exploring potential mediators that could influence the relationship between gut microbiota and RVD. These mediators include immune cells, cardiovascular proteins, and Estradiol. By examining these factors, the research aims to understand how they might interact with gut microbiota to affect the development and progression of RVD, providing a more comprehensive view of the disease’s pathogenesis.

## Method

### Study design

**Figure 1:**
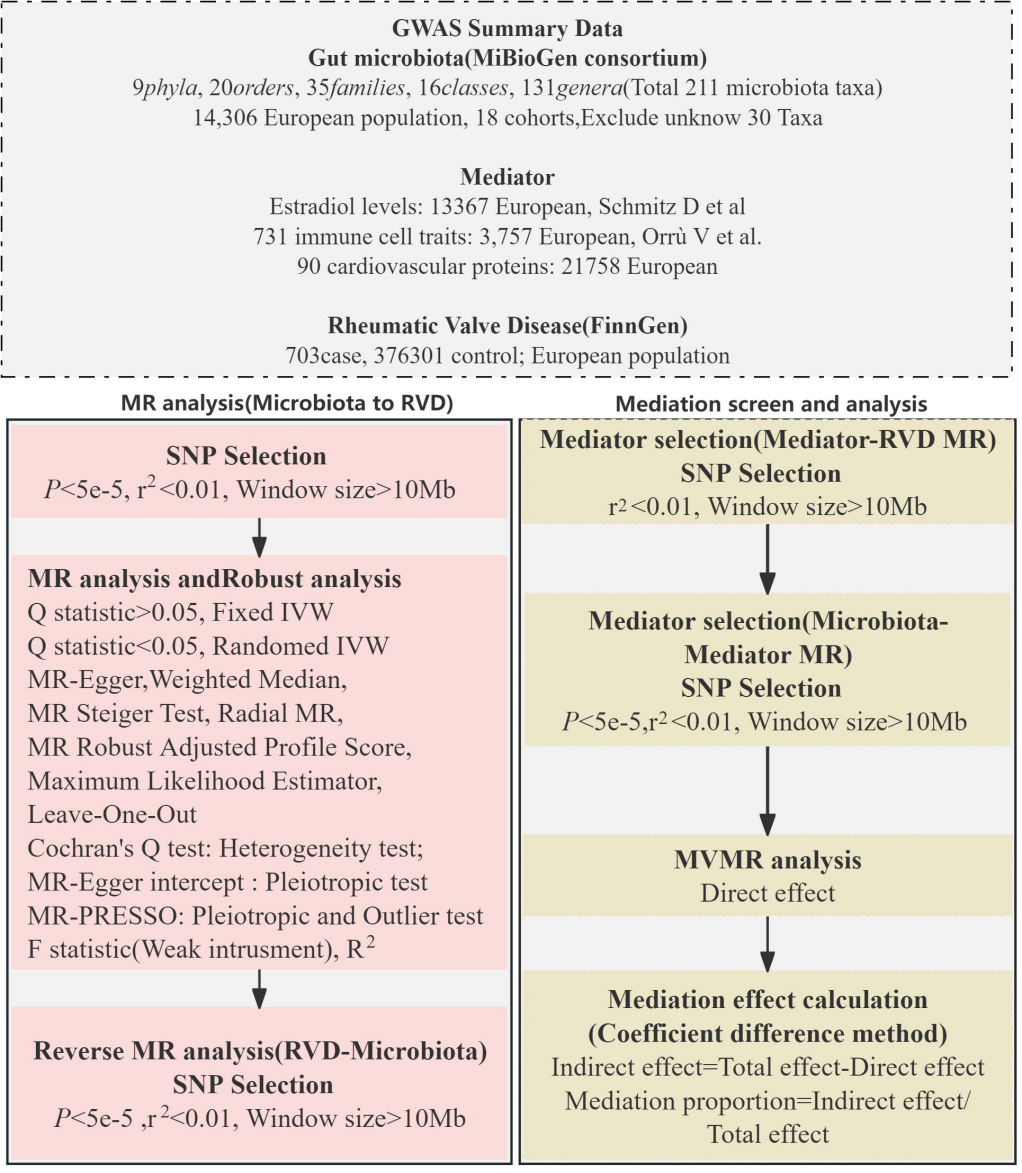
Mendelian randomization flowchart.

Our investigation employed a two-sample MR approach to explore the potential causal connections between gut microbiota and RVD. We adopted a multi-variable MR strategy to enhance our understanding of the mediatory mechanisms involved. The design and progression of our study are detailed in **Figure 1**.

### Data sources

For gut microbiota data, we utilized the extensive dataset from the MiBioGen consortium (12), comprising genome-wide genotypes and 16S fecal microbiome data from 18,340 participants across 24 cohorts. This dataset, primarily from European-descent cohorts, has undergone rigorous adjustments for sex, age, and genetic principal components. Quality control measures were implemented, though external factors like diet and medication were not considered in our analysis. Details can be found in **Supplementary Table 1**. For RVD data, we extracted information from the FinnGen R9 GWAS, which includes 703 cases and 376,301 controls (13).Additionally, datasets from Orrù et al. (14), Folkersen et al. (15), and Schmitz et al. (16) provided insights into immune cell traits, cardiovascular proteins, and Estradiol levels, respectively. All participants in these studies are of European descent. Further information and data link is available in **Supplementary Table 1**.

### Single nucleotide polymorphisms selection

**Figure 2:**
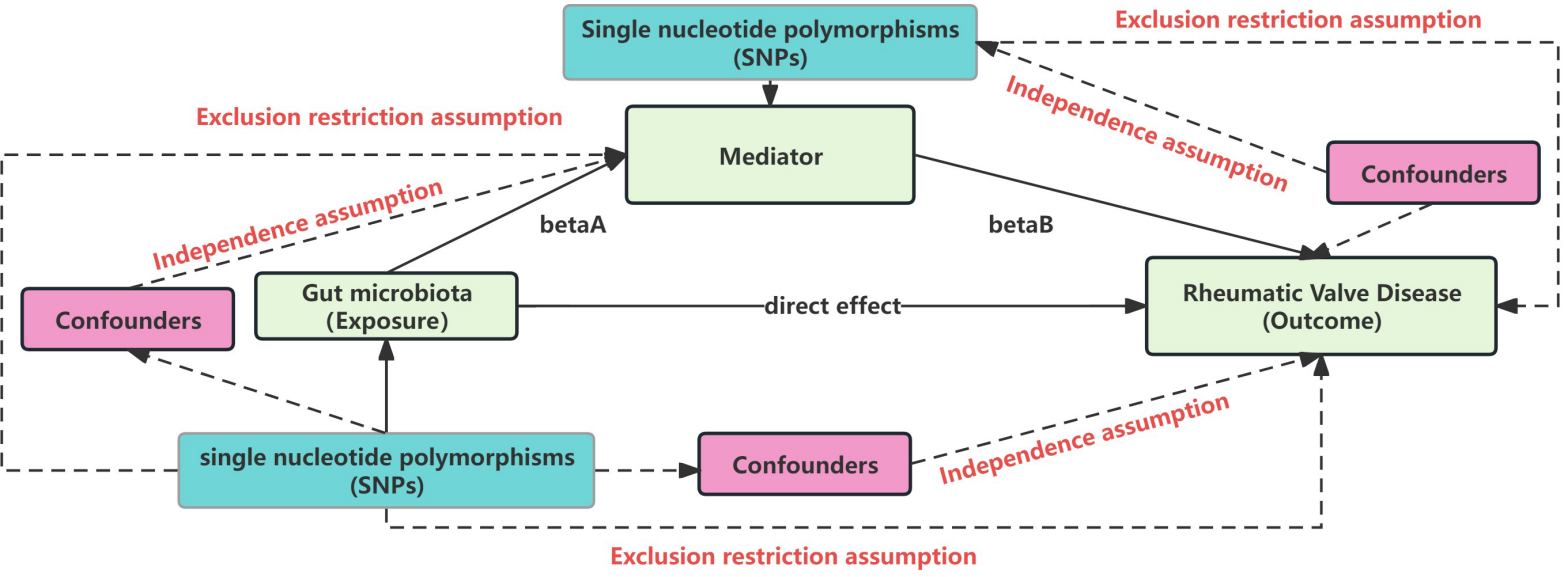
Mendelian randomization core assumption. This Figure represent the MR and mediation assumption, including three core assumption: Independence assumption: SNPs should be robustly associated with the exposure or mediator without confounding factors; SNPs should have a strong link with the exposure or mediator; Exclusion restriction assumption: SNPs should affect the outcome only through the exposure (mediator).

Our Single nucleotide polymorphisms (SNPs) selection adhered to three key MR principles (**Figure 2**) (17): a) Independence assumption: SNPs should be robustly associated with the exposure or mediator without confounding factors; b) SNPs should have a strong link with the exposure or mediator; c) Exclusion restriction assumption: SNPs should affect the outcome only through the exposure (mediator).

We selected SNPs with genome-wide significance (*P* < 5 × 10^−8^) related to exposures initially, then we relaxed the threshold to 5 × 10^−5^ due to a small number of SNPs under *P* < 5 × 10^−8^, *P* < 5 × 10^−7^ and *P* < 5 × 10^−6^. For the reverse analysis focusing on RVD, we selected SNPs with a significance level of *P* < 5 × 10^−5^. In the mediation analysis, SNP selection was further refined by adjusting the *P*-value threshold based on the number of SNPs involved, ensuring a more precise and tailored approach to identifying potential mediators in the relationship between gut microbiota and RVD. Linkage disequilibrium (18) clumping was used to refine SNP selection (r^2^ < 0.01, window size > 10,000 kb), as detailed in **Supplementary Table 2**. We ensured the reliability of these genetic instruments by calculating the F statistic, F = R^2^ × [(N – 1 − k)/k] × (1 − R^2^), where R^2^ represents the variance in the exposure explained by the selected SNPs, N is the sample size, and k denotes the number of SNPs used as instrumental variables. For single SNP, We use Beta^2^_exposure_/SE^2^_exposure_ to calculate the F statistic (19). F value of SNP above 10 indicating sufficient strength to avoid weak instrument bias (20). All of SNP selected can be found in **Supplementary Table 2**.

### Statistical analysis strategy

To investigate the relationships between gut microbiota and RVD, we employed a robust two-sample MR framework. And explore potential mediation including immune cell traits, cardiovascular proteins, and Estradiol levels.

Primary Methodology: Inverse Variance-Weighted (IVW) Method (21): This method aggregates estimates from individual genetic variants to produce a summary causal estimate, assuming all used genetic instruments are valid.

Sensitivity Analysis Methods: Weighted Median (WM) and Simple Median (22): These methods are employed to ensure reliability of our estimates even if some instruments are invalid, with the WM method requiring that at least 50% of instruments are valid. MR-Egger Regression (23): Enables the detection of directional pleiotropy by providing an estimate of the intercept from the regression analysis, which indicates the presence of pleiotropic effects. MR Steiger Test (24): Conducted to ensure the correct direction of causality in genetic associations used in our MR analyses. MR Robust Adjusted Profile Score (MR RAPS) (25): Applied to adjust for pleiotropic effects that may bias the results, enhancing the robustness of causal estimates. Maximum Likelihood Estimator (MLE) (26): This method was used to maximize the statistical efficiency and provide unbiased estimates under the assumption model. Radial MR (27): Offers further investigation into the influence of individual SNPs, ensuring that our causal estimates are not disproportionately affected by any single genetic variant.

Outlier Detection and Heterogeneity Assessment: Leave-One-Out Analysis (28): To assess the impact of each individual genetic variant on the overall MR estimate, we employed the leave-one-out method. This sensitivity analysis involves recalculating the MR estimates repeatedly, each time excluding one genetic variant at a time. This method helps identify whether any specific SNP disproportionately influences the results, thereby ensuring the robustness and stability of our findings. MR-PRESSO (29): Identifies and adjusts for outliers in the genetic instruments to refine the validity of our instrumental variable analysis. Cochran’s Q Test (30): Detects heterogeneity among the estimates provided by different SNPs. We applied a random-effects IVW model when significant heterogeneity was detected.

Multiple Testing Correction: Benjamini-Hochberg Method (FDR) (31): To control the false discovery rate given the multiple comparisons inherent in our analysis, we set a significance threshold of *P* < 0.1. We also noted taxa achieving nominal significance (*P* < 0.05) but not meeting the FDR-adjusted significance threshold (*P*_FDR_ > 0.1) as potentially causal associations.

Software Utilization: All statistical analyses were conducted using R software (version 4.3.1), and the results were visualized through Python-based plotting libraries to ensure clarity and precision.

## Result

### MR analysis between the gut microbiota and RVD

**Figure 3:**
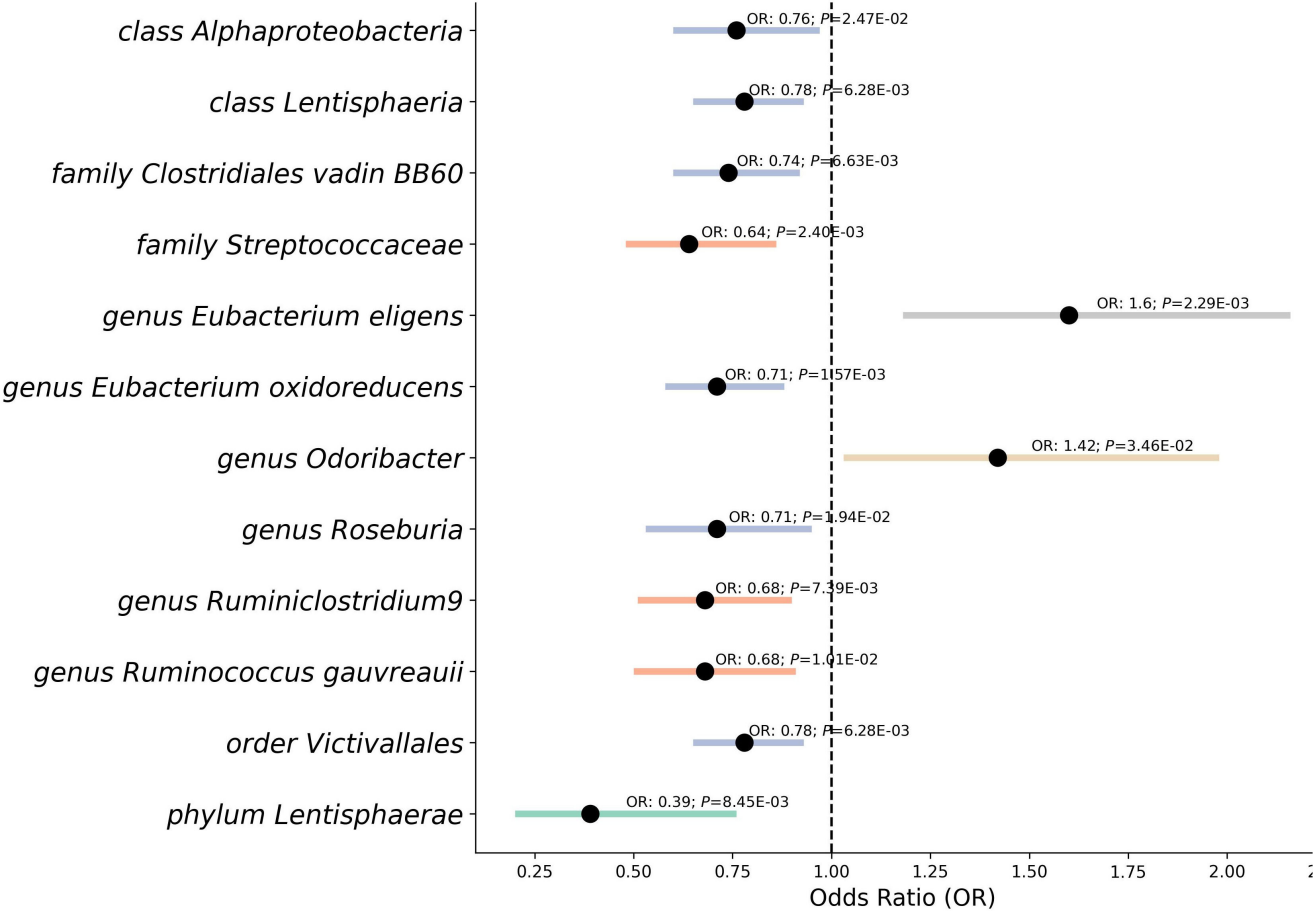
Mendelian randomization analysis between gut microbiota and the risk of RVD. This forest plot represents a Mendelian randomization analysis exploring the association between different classifications of gut microbiota and the risk of Rheumatic Valve Disease (RVD). Each point indicates the Odds Ratio (OR), showing the strength and direction of the association. Horizontal lines represent Confidence Intervals, providing a range for the true OR. Points to the left of the vertical line (OR < 1) suggest a protective effect against RVD, while points to the right (OR > 1) indicate a potential risk. *P*-value denote the statistical significance of each association.

Our study’s Mendelian randomization analysis reveal the relationship between gut microbiota and RVD (**Figure 3**), encompassing a broad range of microbiota. We found that the *phylum*, *class Lentisphaerae* (OR 0.39, *P*=0.008; OR 0.78, *P*=0.006), *classes Alphaproteobacteria* (OR 0.76, *P*=0.025), as well as the *family Clostridiales vadin BB60* (OR 0.74, *P*=0.007), exhibited potential protective effects against RVD. The *family Streptococcaceae* (OR 0.64, *P*=0.002) also showed a similar protective trend. The *genus Eubacterium oxidoreducens* (OR 0.71, *P*=0.002), *genus Roseburia* (OR 0.71, *P*=0.019), and *order Victivallales* (OR 0.78, *P*=0.006) were associated with a reduced risk of RVD.

In contrast, the *genus Eubacterium eligens* (OR 1.6, *P*=0.002) and the *genus Odoribacter* (OR 1.42, *P*=0.035) presented as potential risk factors, indicating an increased likelihood of RVD. Furthermore, our analysis pointed to the *genus Ruminococcus gauvreauii* (OR 0.68, *P*=0.010), *genus Ruminiclostridium9* (OR 0.68, *P*=0.007) suggested protective associations.

After using the MR Steiger Test, we found that no SNPs violated the causal relationship from gut microbiota to RVD. With F-statistics consistently between 14 and 1092 for each SNP, the strength of the genetic instruments used was affirmed. While most of the associations were backed by sensitivity tests like MR Egger, Weighted Median, Simple median, Maximum likelihood, Radial, Raps (**Supplementary Table 3**), and no outliers or pleiotropy were detected by MR-PRESSO analysis (**Supplementary Table 4**). During the leave-one-out sensitivity analysis (**Supplementary Table 5**), we identified that the *genus Ruminococcus1*, *class Bacilli* had some SNP outlier, and Radial, Raps analysis can not support their significant casual relationship with RVD, which may potentially distort the causal estimate. Then we used LDtrait (32) found 100 unique SNPs (**Supplementary Table 6**) with significant associations with other traits, raising the possibility of pleiotropy or confounding. We reanalyzed our data after removing the 100 SNPs and results of this reanalysis are presented in **Supplementary Table 7**. The associations between *genus Ruminococcus* and *class Bacilli* with RVD were also no longer significant. After careful consideration, we have decided to exclude these two exposure. The *phylum Lentisphaerae* did display pleiotropy, leading us to adopt the MR-Egger method as the main result for this taxa. These results post-FDR adjustment revealed that several GM taxas remain significant: *Class Lentisphaeria*: *P*_FDR_ = 0.094; *Family Clostridiales vadin BB60*: *P*_FDR_ = 0.099; *Family Streptococcaceae*: *P*_FDR_ = 0.072; *Phylum Lentisphaerae*: *P*_FDR_ = 0.084. Other results are considered as potentially causal associations.

**Figure 4:**
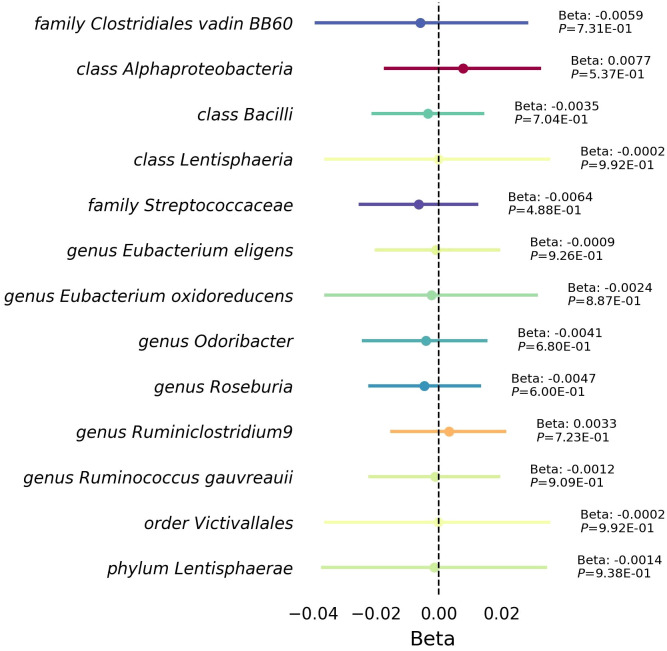
Mendelian randomization analysis between RVD gut microbiota. This forest plot illustrates a statistical analysis investigating the relationship between Rheumatic Valve Disease (RVD) and gut microbiota. Each horizontal line represents a different microbial classification, with the central dot indicating the beta coefficient (effect size). The span of each line shows the 95% confidence interval, indicating the range within which the true effect size is likely to fall. A beta coefficient to the right of the zero line (positive value) suggests a potential increase microbiota abundance with RVD, while a coefficient to the left (negative value) indicates a potential decrease microbiota abundance with RVD. The annotations to the right of each line provide the beta coefficient and *P*-value, with the *P*-value indicating the statistical significance of the association.

Furthermore, our reverse MR analysis indicated a no significant association between RVD and these 12 microbiotas (**Figure 4**, **Supplementary Table 8**).

### Mediators selection

**Figure 5:**
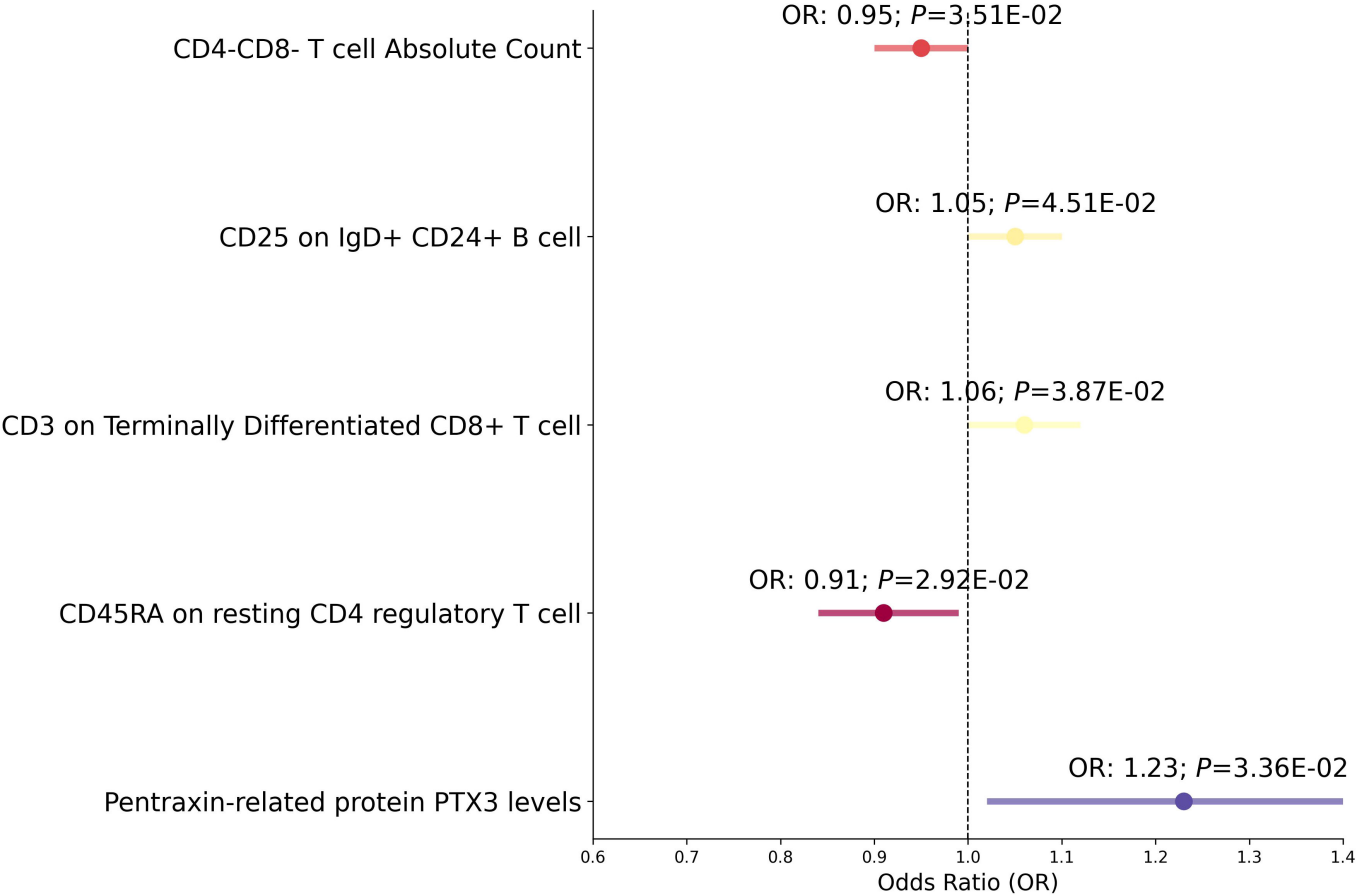
Mediation analysis between Mediator and RVD. This forest plot illustrates a statistical analysis investigating the relationship between various immunological markers and hormone levels with the risk of RVD. Each horizontal line represents the odds ratio (OR) for RVD risk. An OR below 1 suggests a potential protective effect, while an OR above 1 indicates a potential increased risk. The span of each line shows the 95% confidence interval, indicating the range within which the true effect size is likely to fall. The annotations above each line provide the OR and *P*-value, with the P-value indicating the statistical significance of the association.

In our Mendelian randomization study, we first examined the potential mediators including immune cell traits, cardiovascular proteins, and Estradiol levels to ascertain their effect on RVD (**Figure 5**, **Supplementary Table 9**). The analysis revealed several mediators with significant associations: CD4^-^CD8^-^ T cell Absolute Count, CD25 on IgD^+^CD24^+^ B cells, CD3 on Terminally Differentiated CD8^+^ T cells, CD45RA on resting CD4 regulatory T cells, Pentraxin-related protein PTX3 levels, and Estradiol levels all showed varying degrees of association with RVD, with odds ratios ranging from 0.91 to 1.23 and *P* from 0.0292 to 0.0451. After removing confounding SNPs, The associations between Estradiol levels and RVD were no longer significant(**Supplementary Table 9**).

**Figure 6:**
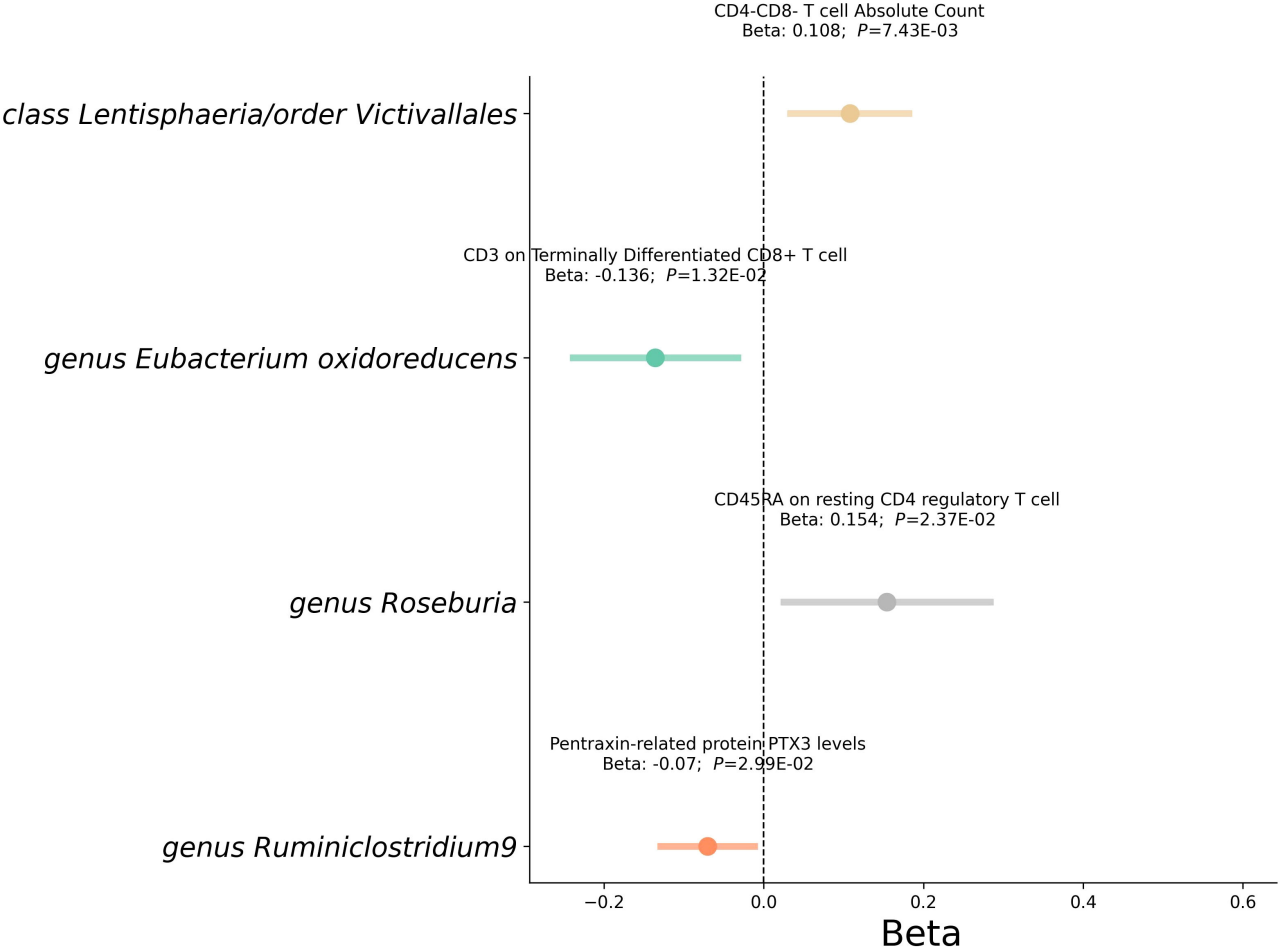
Mediation analysis between gut microbiota and Mediator. This forest plot illustrates a statistical analysis investigating the relationship between gut microbiota and mediator. Each horizontal line represents the effect of microbial classification on mediator, with the central dot indicating the beta coefficient (effect size). The span of each line shows the 95% confidence interval, indicating the range within which the true effect size is likely to fall. A beta coefficient to the right of the zero line (positive value) suggests a potential increase in RVD risk with that mediator, while a coefficient to the left (negative value) indicates a potential decrease in risk. The annotations above each line provide the beta coefficient and *P*-value, with the P-value indicating the statistical significance of the association.

Subsequent analysis delved into how gut microbiota influences these mediators (**Figure 6** and **Supplementary Table 10**). Notably, the *class Lentisphaeria* was positively associated with CD25++ CD45RA+ CD4 not regulatory T cell %CD4+ T cell and CD4^-^CD8^-^ T cell Absolute Count. *Genus Roseburia* increased CD45RA expression on resting CD4 regulatory T cells. Similarly, the *order Victivallales* showed positive associations with CD25++ CD45RA+ CD4 not regulatory T cell %CD4+ T cell and CD4^-^CD8^-^ T cell counts. In contrast, *genus Eubacterium oxidoreducens* and *genus Ruminiclostridium9* demonstrated an inverse relationship with CD3 on Terminally Differentiated CD8+ T cell and PTX3 levels respectively.

### Mediation effect

The multivariable Mendelian randomization analysis in our study elucidated the intricate mediating role of immune cells and biomarkers in the association between gut microbiota classes and RVD (**Table 1**). *Class Lentisphaeria*, as well as the *order Victivallales*, showed notable mediation effects through immune cell proportions and counts, with mediation effects of 18.17%. The *genus Eubacterium oxidoreducens* displayed a direct influence on RVD, moderated by CD3 on Terminally Differentiated CD8^+^ T cells, respectively, with mediation effects above 11%. Interestingly, *genus Roseburia* and *genus Ruminiclostridium9* also emerged as significant players, with mediation effects of 13.86% and 37.57% through CD45RA on resting CD4 regulatory T cells and Pentraxin-related protein PTX3 levels, respectively.

**Table 1:** Multivariable Mendelian randomization analysis among Gut microbiota, Mediator and RVD.

Exposure	Mediator	Outcome	Total effect	Direct effect	Mediation effect
*class Lentisphaeria/order Victivallales*	CD4-CD8- T cell Absolute Count	RVD	-0.247	-0.202	18.17%
*genus Eubacterium oxidoreducens*	CD3 on Terminally Differentiated CD8+ T cell	RVD	-0.338	-0.300	11.23%
*genus Roseburia*	CD45RA on resting CD4 regulatory T cell	RVD	-0.342	-0.295	13.86%
*genus Ruminiclostridium9*	Pentraxin-related protein PTX3 levels	RVD	-0.384	-0.240	37.57%

## Discussion

In the discussion of our findings from the Mendelian randomization analysis, we reflect on the complex interactions between gut microbiota and RVD. Our study identifies 12 taxa (include 1 *phylum*, 1 *order*, 2 *class*, 2 *family*, 6 *genus*) within the gut microbiome that appear to exert a protective effect against RVD, such as the *class Lentisphaeria* and the *family Clostridiales vadin BB60*. These associations are supported by robust statistical analyses, with F-statistics indicating strong instrument strength and sensitivity analyses confirming the reliability of our findings.

The selection of mediators such as immune cell types and cardiovascular proteins revealed significant associations with RVD, suggesting that the effects of the gut microbiota on RVD may be mediated through these biological pathways. Our multivariable MR analysis confirmed the mediating roles of these factors, with certain microbiota classes showing substantial mediation effects, such as the *genus Ruminiclostridium9*’s impact on PTX3 levels.

These findings underscore the potential for gut microbiota to affect the immune system and inflammatory processes, contributing to the development or exacerbation of RVD. The mediation effects observed, ranging from 11% to over 37%, indicate that a significant portion of the microbiota’s influence on RVD may be channeled through these immune and inflammatory mediators.

The intestinal microbiota, a complex and diverse community of microorganisms, plays a crucial role in human health. This ecosystem contains over 1,500 different species of bacteria, predominantly from the *phyla Bacteroidetes* and *Firmicutes*, which together make up about 90% of the gut microbial community (33). These microorganisms are essential for various bodily functions, including digestion and immune system support.

One of the key roles of the gut microbiota is to protect the host against pathogens (34). This is achieved through various mechanisms, including colonizing mucosal surfaces and producing microbial metabolites. These metabolites can inhibit the growth of harmful bacteria and contribute to the overall health of the host. Apart from the gut, the second most complex microbial community in the human body is found in the oral cavity (35). This community is not only important for oral health but also has a significant impact on systemic health. The metabolites produced by the gut microbiota, including SCFA like acetate and butyrate, amino acid derivatives such as indole, bioactive gases, bile acid transformations, polyamines, vitamins, bioactive peptides, and endocannabinoids, play essential roles in maintaining physiological homeostasis (36). These metabolites impact various health outcomes, influencing metabolic processes, immune function, and gut-brain communication.

Within the human gut microbiome, the *class Lentisphaeria* and the *order Victivallales*, part of the *Lentisphaerae phylum*, are primarily represented by the species *Victivallis vadensis (* 37). Notably, *Victivallis vadensis* is known for its unique production of acetate and formate (38). Our study associates these microbiota with a reduced risk of RVD. The mediation analysis suggests a possible mechanism: these microbes might increase the count of CD4^-^CD8^-^ (double-negative, DN) T cells, which are known to have both innate and adaptive immune functions, distinct from conventional CD4^+^ and CD8^+^ T cells. DN T cells, despite comprising only 3–5% of T lymphocytes in peripheral blood, have demonstrated their potential in regulating immune responses. This includes suppressing activated T cells, B cells, and dendritic cells in various mouse models (39–42). The increase in DN T cells, potentially influenced by acetate and formate from *Lentisphaeria* and *Victivallales*, could contribute to the observed reduced risk of RVD. Supporting this, research by Park et al. (43) emphasizes acetate’s significant role in T lymphocyte proliferation, modulated by various cytokines and immune factors. Acetate is particularly effective in enhancing the population of IL-10 producing T lymphocytes, which are crucial for their anti-inflammatory properties. This effect of acetate aligns with our findings, suggesting that the metabolites produced by *Lentisphaeria* and *Victivallales*, particularly acetate, may contribute to the modulation of immune responses, thereby impacting the risk and progression of inflammatory conditions like RVD.

Butyrate production in the gut, crucial for colon health, is primarily facilitated by specific bacterial species within the *Ruminococcus*, *Faecalibacterium*, *Eubacterium*, and *Roseburia genera*. These bacteria play a key role in synthesizing butyrate, a vital SCFA in the gastrointestinal tract (44, 45). Butyrate is particularly influential in T cell differentiation and function, notably enhancing the presence and activity of regulatory T cells (Tregs) in the gut (46). Tregs are crucial for maintaining immune tolerance and preventing autoimmune disorders (47). In the context of RVD, our study finds a correlation between the presence of certain gut microbiota and a decreased risk of RVD. Specifically, *genera* such as *Ruminiclostridium9*, *Ruminococcus gauvreauii*, and *Eubacterium oxidoreducens* show this association. Our mediation analysis further suggests that *Eubacterium* can reduce the expression of CD3 on terminally differentiated CD8^+^ T cells. Given that CD3 is integral to the T-cell receptor (TCR) complex and essential for TCR signaling and T cell activation (48), this reduction could influence the immune response related to RVD.

Additionally, our analysis indicates that *Ruminiclostridium9* can lower levels of pentraxin PTX3. PTX3, part of the long pentraxin subfamily and inducible by IL-1 or TNF, plays a role in infection defense, tissue repair, and managing inflammation in cancer (49, 50). It’s also implicated in various cardiovascular diseases, including heart failure, atherosclerosis, acute coronary syndrome, peripheral vascular diseases, and rheumatic mitral valve stenosis (51–54). PTX3’s involvement extends to the breakdown of fibrin-rich deposits at injury sites and the subsequent collagen deposition (55). This data suggests a multifaceted influence of the gut microbiota on RVD risk, where specific bacterial genera not only affect the butyrate levels and thus Treg function but also modulate key immune mediators like CD3 and PTX3, which are critical in cardiovascular health and immune response regulation.

RVD is primarily characterized by an autoimmune response involving CD4^+^ T helper cells, which play a central role in orchestrating the immune response (56). These cells activate other immune cells, such as B cells and macrophages, potentially leading to inflammation and subsequent damage to the heart valves (57, 58). Within the human CD4^+^ T cell population, there are two distinct subpopulations with different phenotypes and functions: CD45RA^+^ resting regulatory T cells (rTreg cells) and CD45RA^−^ activated regulatory T cells (aTreg cells). CD45RA^+^ rTreg cells, upon stimulation, differentiate into CD45RA^−^ aTreg cells and proliferate (59). Given the rapid turnover of aTreg cells and the robust proliferative ability of rTreg cells upon activation, therapeutic strategies aimed at expanding Treg cells ex vivo should prioritize rTreg cells, as supported by existing research. Our mediation analysis reveals that the *genus Roseburia* increases the levels of CD45RA in resting CD4 regulatory T cells. This finding is particularly significant for managing long-term inflammation in RVD. By potentially enhancing the rTreg cell population, *Roseburia* may contribute to a more controlled and balanced immune response, thereby mitigating the progression and severity of inflammation in RVD. This underscores the importance of understanding gut microbiota’s influence on specific immune cell subsets, especially in the context of autoimmune diseases like RVD.

Our study interestingly suggests that the *Streptococcaceae family* may play a role in reducing the risk of RVD. This observation aligns with the absence of *Streptococcus hemolyticus*, a pathogenic bacterium associated with RVD, in the normal human gut microbiome. Our GWAS data from the European population, which did not report RVD patients, supports the hypothesis that beneficial members of the *Streptococcaceae family*, such as *Streptococcus thermophilus*, commonly found in the human gut (60), might contribute to this protective effect against RVD.

Our study distinguishes itself through its thorough and multi-faceted approach, meticulously examining the links between gut microbiota and RVD. Our methodology’s strength lies in the use of diverse, rigorous analytical techniques including the weighted median, MR-Egger, IVW method, MR Steiger Test, MR RAPS and Leave-One-Out, all of which contribute to the reliability of our findings. Additionally, the implementation of the MR-PRESSO strategy enhances the validity of our results by identifying and correcting for potential outliers, thus reducing bias. A notable aspect of our research is the in-depth investigation of specific genera within the gut microbiota and their correlation with RVD. Although some correlations became statistically insignificant after adjusting for multiple tests, we maintain a focus on uncovering as many potential associations as possible, accepting the risk of encountering false positives. These findings offer valuable insights into possible biological interactions. Another strength of our study is the homogeneity of our sample population, which predominantly consists of individuals of European descent. This uniformity helps to minimize variations due to population differences, adding another layer of consistency to our research.

However, our research does have certain limitations. The most significant of these is the reliance on data from European populations, which could introduce biases and limit the generalizability of our results to other ethnic groups. Additionally, the absence of individual-level data limited our ability to delve into more complex relationships, possibly leading to an oversight of non-linear associations between the gut microbiota, immune cell traits, and RVD. Therefore, specific patterns of association, like U-shaped or J-shaped relationships, might not have been fully captured in our study.

Overall, our study adds to the growing body of evidence that gut microbiota are intricately linked to systemic diseases such as RVD. It points to the importance of understanding the microbiome’s role in disease mechanisms, which could pave the way for novel therapeutic strategies that target the microbiome to modulate disease risk and progression.

## Future research

Future research should pivot towards a multifaceted approach. This includes expanding the scope to include a broader range of ethnic groups for a more comprehensive understanding of microbiota variations across different demographics. A deeper analysis of how specific bacteria and their metabolites, such as acetate and butyrate, influence immune responses is crucial. Investigating therapeutic interventions like diet modifications or probiotics can reveal how changes in the gut microbiome impact RVD risk and progression. Personalized microbiome-based treatment strategies, tailored to individual microbiome compositions, could be a breakthrough in RVD prevention and management. Long-term studies are essential to track gut microbiota changes over time and their correlation with the development of RVD. Employing advanced sequencing and data analysis techniques will uncover novel microbial species and complex interactions within the microbiome. Lastly, public health education focusing on the importance of gut health and its broader impact on diseases like RVD is vital. Such a comprehensive and integrated research approach will pave the way for more effective strategies in understanding and managing RVD.

## Data availability statement

The original contributions presented in the study are included in the article/**Supplementary Material**. Further inquiries can be directed to the corresponding authors.

## Ethics statement

The data utilized for this study are accessible in the public data, have received ethical approval, and were collected with informed consent from the participants.

## Author contributions

XC: Writing – original draft, Methodology, Investigation, Data curation, Conceptualization. GH: Writing – original draft, Methodology, Investigation, Data curation, Conceptualization. DN: Writing – review & editing, Validation, Supervision, Software, Methodology, Formal analysis. DW: Writing – review & editing, Validation, Supervision, Resources, Funding acquisition.
